# Fate and Transport of Polycyclic Aromatic Hydrocarbons in Upland Irish Headwater Lake Catchments

**DOI:** 10.1100/2012/828343

**Published:** 2012-12-31

**Authors:** Heidi E. M. Scott, Julian Aherne, Chris D. Metcalfe

**Affiliations:** Environmental and Resource Studies, Trent University, Peterborough, ON, Canada K9J 7B8

## Abstract

Polycyclic aromatic hydrocarbons (PAHs) are a concern due to their carcinogenicity and propensity for transboundary atmospheric transport. Ireland is located on the western periphery of Europe and assumed to receive clean Atlantic air. As such, it has been used as an atmospheric reference for comparison to other regions. Nonetheless, few studies have evaluated concentrations of PAHs within the Irish environment. In the current study, PAHs were measured at five upland (500–800 masl) headwater lake catchments in coastal regions around Ireland, remote from industrial point source emissions. Semipermeable membrane devices were deployed in lakes for a 6-month period in July 2009, and topsoils were sampled from each catchment during October 2010. The concentrations of PAHs were low at most study sites with respect to other temperate regions. Homologue groups partitioned between lake and soil compartments based on their molecular weight were: “lighter” substances, such as Phenanthrene and Fluorene, were found in higher proportions in lakes, whereas “heavier” compounds, such as Chrysene and Benz[a]anthracene, were more prominent in soils. Concentrations of PAHs were highest at the east coast sites, potentially due to contributions from historical transboundary and regional combustion sources.

## 1. Introduction

Polycyclic aromatic hydrocarbons (PAHs) is an overarching term describing hundreds of individual chemical compounds containing two or more fused aromatic rings and are known to persist or accumulate in the environment [1]. Although PAHs occur naturally in the environment (e.g., forest fires, volcanoes, and diagenesis), their natural cycle has been significantly augmented during the past century through anthropogenic processes, such as wood and fossil fuel combustion [2–7]. Combustion is the primary source of long-range atmospheric transport of PAHs into the surrounding environment [8]. However, unlike many legacy persistent organic pollutants (POPs), that typically follow the global distillation model of accumulation in colder regions owing to atmospheric condensation and cold trapping [9], PAHs tend to decrease in concentration further from the initial point source [1]. Atmospheric concentrations of PAHs tend to be higher in temperate regions owing to seasonal heating [10]. In addition, the Arctic still exhibits preindustrial levels of PAHs (e.g., 1–10 ng g^−1^ per individual PAH [1, 11]) largely owing to the lower atmospheric half-life [12] caused by thermal/photodegradation and propensity for particulate binding [13]. Further, regions receiving high rates of precipitation are particularly vulnerable due to the dominant “washout” of contaminants from the atmosphere [14].

 The ubiquitous nature of PAHs in the environment, primarily stored in soils [1], is a major human and ecosystem health concern owing to their known carcinogenicity and potential toxicity to both aquatic and terrestrial organisms [15, 16]. Observations of PAHs in regions remote from direct emission or production provide an understanding of the influence of atmospheric transport on ecosystem integrity. Ireland is a well established background reference region for atmospheric research owing to its location on the western periphery of Europe and dominant prevailing Westerlies [17]. West coast sites are characterised by clean oceanic air while east coast sites show an anthropogenic signal from national and transboundary sources. However, there is limited knowledge on PAHs in the Irish environment; research to date has focused only on lake sediment cores at a few sites [18].

 The objective of this research was to provide a comprehensive assessment of PAHs in seminatural upland headwater lake catchments in Ireland, dominated by loadings from atmospheric deposition, catchment response, and recycling from sediments. The level of PAHs in lake water and surrounding soils was quantified in upland catchments (*n* = 5) remote from primary industrial emission sources. In addition, the variation between sites and proportions of individual PAHs was evaluated. Source apportionment and tentative relationships between PAHs and various physical, chemical, and meteorological parameters were also evaluated to assess sources and fate.

## 2. Materials and Methods

### 2.1. Study Sites

**Figure 1:**
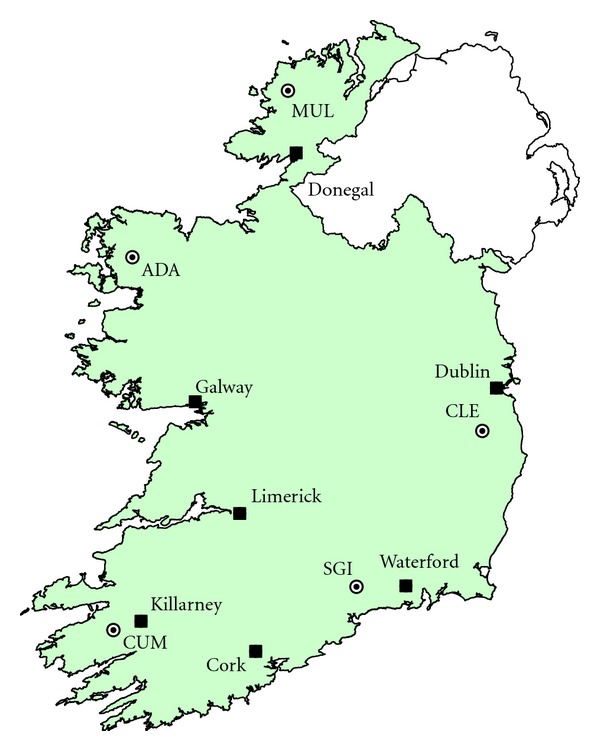
Location of the five study sites (circles) and major cities (filled squares) in the Republic of Ireland (green shading): Lough Cummeenoughter (CUM), Lough Adanacleveen (ADA), Mullincrick Lough (MUL), Sgilloge Lough (SGI), and Cleevaun Lough (CLE).

The five headwater lake catchments (Lough Cummeenoughter (CUM), Lough Adanacleveen (ADA), Mullincrick Lough (MUL), Sgilloge Lough (SGI), and Cleevaun Lough (CLE)) were located in upland regions (Figure 1, Table 1) and represented a transboundary network analogous to the atmospheric monitoring network established by the Irish Environmental Protection Agency under the European Monitoring and Evaluation Programme [19]. The study sites were located in acid-sensitive moorland regions [20]; the catchments were dominated by organic soils and the vegetation was primarily *Calluna vulgaris* along with various graminoid (e.g., sedges and grasses) and bryophyte (e.g., mosses and lichens) species. The catchments ranged in size from 1.90 to 63.4 ha (mean: 19.1 ha) and elevation ranged from 493 to 713 masl (mean: 590 masl). The lakes varied in size from 0.35 to 2.36 ha (mean: 1.37 ha) and were polymictic (well-mixed) due to their shallow nature (depth 1–8 m) and the relatively mild and windy climate. Annual wind speeds are on average 11 km hr^−1^ in the south and up to 29 km hr^−1^ in the north [17]. Long-term annual rainfall at the study sites ranged from 1600 to 3000 mm and mean air temperatures ranged between 9 and 10.3°C (Table 1) based on Met Éireann (The Irish Meteorological Service) 1960–1990 climate normals [21]. Pollutant inputs were assumed to be predominantly deposited from the atmosphere owing to their remoteness from local pollution sources.

### 2.2. Field Sampling

Water samples for major ion chemical analysis were collected from the shore of each lake in 250 mL plastic high density polyethylene bottles at regular intervals during the study period (July 2009–July 2011; *n* = 8), at a depth of not more than 20 cm below the surface in areas free of debris and vegetation. Each bottle was rinsed with lake water five times prior to sample collection and filled and capped underwater to avoid headspace. Supplementary physical and chemical lake parameters measured on site included water temperature, pH, conductivity, and dissolved oxygen (DO). In addition, continuous hourly water temperatures were monitored using HOBO pendant loggers.

 The concentration of PAHs in lakes was monitored using semipermeable membrane devices (SPMDs) immersed in the lakes. Semipermeable membrane devices are passive samplers used to monitor the presence of lipophilic organic contaminants at concentrations as low as parts per trillion (i.e., ng L^−1^; [22]). These devices only measure true dissolved aqueous phase organic pollutants (and do not absorb compounds bound to organics or particulate matter), available for partitioning into synthetic lipid (triolein), given that the target pollutants are generally hydrophobic and lipophilic (*K*_ow_ > 4.4). The SPMDs were assembled in a “Class A” clean laboratory at Trent University [23]. The SPMDs contained 1 mL of high purity (95%) triolein in polyethylene bags and were prepared with performance reference compounds (PRCs): 30 ng of PCB congener 14, 32, and 155, and 25 ng of congener 203, which were used to assess the influence of environmental conditions on SPMD function [24]. Prior to deployment, three SPMDs were suspended inside a cylindrical shroud (77 × 10 cm) and installed in each study lake. The shrouds were constructed from galvanized metal stove pipe with holes punched to allow for water movement. The SPMDs (*n* = 3 per site) were submerged at a depth of ~1 m in each lake during July 2009 and retrieved in January 2010. Upon recovery, the SPMDs were placed back into their initial solvent-washed jars and stored in a dark cooler until they could be refrigerated. In the laboratory, SPMDs were cleaned using deionized water and separated from solution using hexane [25]. Trip blanks were exposed to air on-site during each deployment and a laboratory blank was left at Trent University, both were used to correct for background contamination.

 High molecular weight (HMW) PAHs (>4 aromatic rings) possess a strong affinity (binding) for organic matter and do not partition readily into SPMDs [26]; however, they can be monitored through catchment soil sampling. As such, soils were collected at each study site and analyzed for PAHs. Prior to sampling, all equipment was washed with Sparkleen and rinsed several times with deionized water. Reagent grade hexane and acetone were used on all equipment surfaces to ensure residues were removed prior to sampling. Soil samples were gathered with a stainless steel corer from topsoils (0–5 cm) following removal of the upper portion of vegetation [27]. The first two core samples were discarded and the subsequent three soil cores were placed in a stainless steel tray and blended into a uniform mixture. The bulk sample was placed into a 125 mL solvent-washed amber glass jar and sealed in a Ziploc bag. Jars were immediately placed in a dark cooler until they could be refrigerated and freeze dried for subsequent analysis. In the laboratory, PAHs were extracted from the soils using accelerated solvent extraction (ASE). A replicate core with a known volume was also taken at each site to determine bulk density in conjunction with additional soil samples (0–5 cm) for supplementary analysis (e.g., pH, organic matter, etc.). Continuous soil temperature at a depth of ~10 cm was monitored during the study period using a buried HOBO pendant logger. 

### 2.3. Laboratory Analysis

In the laboratory, water samples were analyzed for a suite of water chemistry parameters: pH was measured by low conductivity electrode, alkalinity was determined using a PC titration Plus System, total organic carbon (TOC) was analyzed on a TOCV-cph Shimazdu Analyzer, absorbance (ABS) was measured using a UV-VIS spectrometer, and chloride (Cl^−^) and sulphate (SO_4_^2−^) ions were analyzed on an Dionex Ion Chromatograph. Oxygen18 (*δ*18O) and deuterium (*δ*D) were measured with a ThermoFinnigan TC/EA Delta Plus XL Mass Spectrometer at the University of Saskatchewan and used to calculate deuterium excess (d-excess [28]), which has been shown to differentiate lake catchments characterized by slow-moving waters and high evaporation rates versus those with regular throughflow [29].

Soils were oven-dried at 60°C for 48 hours, sieved (<2 mm), and analyzed for total carbon, nitrogen, and sulphur content using a Vario MACRO-CNS Analyzer (Elementar Americas, Inc., NJ, USA), soil organic matter (SOM) was determined as loss-on-ignition (LOI) using a muffle furnace at 550°C for 8 hours, bulk density was calculated based on known volume and dry weight of the core samples, and pH was measured in distilled water using an OakTon pH/mV meter (OakTon Instruments, IL, USA).

 The soil and SPMD extracts were cleaned up by gel permeation chromatography followed by fractionation via silica gel chromatography [25] to isolate PAHs. The samples were subsequently evaporated and redissolved in iso-octane prior to analysis [30]. The SPMD and soil extracts were analyzed for 15 “priority” PAHs (defined by the US EPA) using gas chromatography with low resolution mass spectrometry (GC-LRMS; [26]). Chrysene and Benz[a]anthracene were coeluted during GC-MS analysis, as such they are presented together.

### 2.4. Source-Receptor Analysis

**Figure 2:**
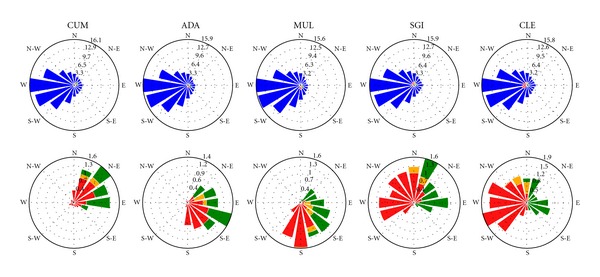
Upper: wind rose source-receptor plots showing the proportion (%) of air by direction and source (Republic of Ireland (red), Northern Ireland (orange), Great Britain (green) and marine and other regions (blue)) arriving at the study catchments (receptors; arrival height of 850 hPa) based on two-day back trajectories estimated every six hours during the period 1989–2009.

The atmospheric source regions for the study sites were evaluated using back-trajectory analysis for one, two, three, and five days based on a simple single particle Lagrangian backtracking algorithm. The analysis focused on estimating the proportion of air during each back-trajectory period (1–5 days) that crossed source region land masses (e.g., Europe (EUR), Great Britain (GB), Northern Ireland (NI), Republic of Ireland (ROI), North America, etc.) before arriving at the study sites (i.e., receptors; arrival height of 850 hPa). Further, trajectory source regions were separated into 8 and 16 directional sectors (i.e., north, north east, east, etc.). Back trajectories were estimated every six hours during the period 1989–2009 using historical wind fields (based on observed data and model output) smoothed onto a 3-dimensional grid with 16 pressure levels and a horizontal resolution of 1 × 1 degree obtained from the ECMWF ERA Interim data set [31]. In this study, “1-day ROI+NI” was defined as the percentage of air originating from the Republic of Ireland and Northern Ireland based on a 24-hour back trajectory, “1-day GB+EUR” as the percentage of air originating from Great Britain and Europe based on a 24-hour back trajectory, “5-day Marine” as the percentage of air originating over oceanic regions based on a 120-hour back trajectory, and “1-day overland” as air originating from the Republic of Ireland, Northern Ireland, Great Britain, and European landmasses based on a 24-hour back trajectory (Figure 2).

### 2.5. Data and Statistical Analysis

Average time-weighted water concentrations were estimated from measured SPMD data based on models discussed in detail by [32], and more recently by [33, 34]. Estimates accommodated PRC data, chemical uptake rates, exposure time, quantity of chemicals measured in SPMDs, and specific octanol water partition coefficients [24]. Version 5.1 of the SPMD Water Concentration Estimator [35] were used to produce estimates of water concentrations from raw SPMD data, which were calculated as average-time weighted totals based on the bulked average of three SPMD samples. Total PAHs in soils were expressed as concentration per weight of soil (ng g^−1^) and by sample mass (i.e., pool of PAHs (*μ*g m^−2^)), estimated using soil bulk density and sample depth (5 cm). All data were tested for normality (Shapiro-Wilk). Pearson Product-Moment Correlation coefficients were used to evaluate correlations between variables and considered to be significant at *P* < 0.05. All correlations were visually assessed for outliers or skewed relationships. The limitations of statistically evaluating the tentative relationships of a small sample size (*n* = 5) using parametric procedures were outweighed by the insight offered from the analysis. Although many parameters were included in the analysis, only statistically significant results are presented. All statistical analyses were carried out using SigmaPlot 11.0 (Systat Software, Inc.). Principal component analysis (PCA) was carried out using Multibase, an Excel add-in program; a PCA is a graphical statistical analysis tool used to process and visually simplify large sets of data allowing the relationships between PAHs and a wide number of physical, chemical, and meteorological site variables (e.g., lake : catchment ratio, pH, organic matter, rainfall, etc.) to be explored. Only components with eigenvalues >1 were retained to uphold reliability of the final output [36].

## 3. Results and Discussion

### 3.1. PAHs at the Study Sites

**Figure 3:**
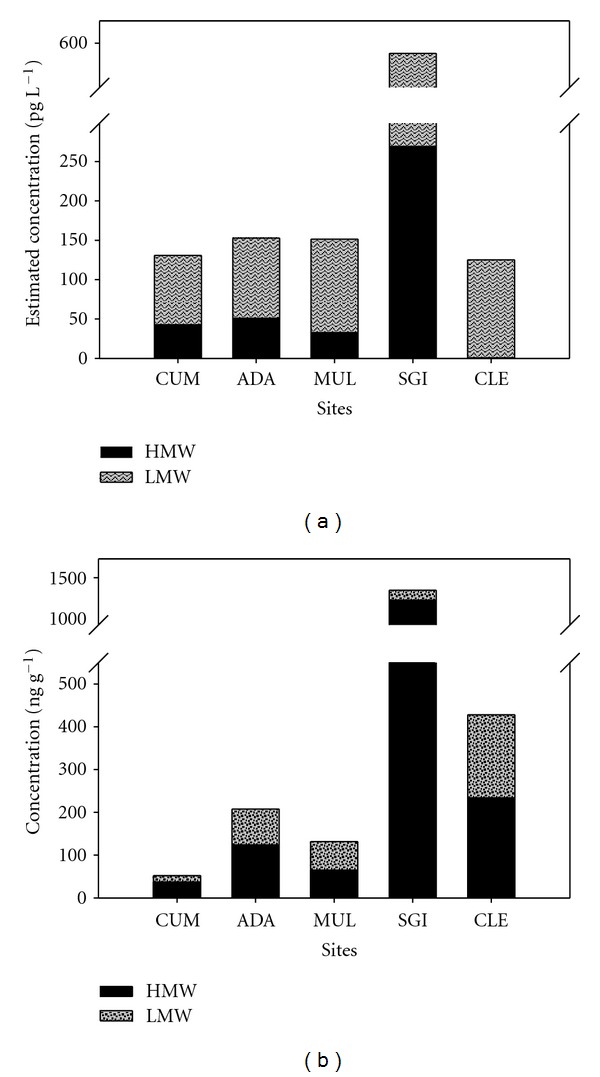
Sum of 15 PAH compounds in the five study lakes (Lough Cummeenoughter (CUM), Lough Adanacleveen (ADA), Mullincrick Lough (MUL), Sgilloge Lough (SGI), and Cleevaun Lough (CLE)) with high molecular weight (black) and low molecular weight (grey) portions shown. Concentrations of PAHs in lakes (a) estimated from SPMDs deployed during the period from July, 2009, to January, 2010. Concentrations of PAHs in soils (b) collected in October 2010.

In general, low molecular weight (LMW) PAHs (2-3 rings) were dominant in the lakes (Figure 3), whereas HMW PAHs (>4 rings) contributed a larger proportion in the soils. The highest concentration of PAHs in water and soil compartments was found at SGI (east coast; see Figure 1). The levels at SGI were considerably elevated compared with the other study sites, highlighting its proximity to historic industrial emissions, and present-day agriculture sources, as evidenced by burning of heathland to promote grass growth, a significant source of PAHs to surrounding ecosystems [37, 38]. Previous studies have similarly observed higher concentrations of PAHs in sediment cores from an east coast upland lake (close to CLE) attributed to elevated historic inputs with maximum levels coinciding with European SO_2_ peaks [18]. The differences in observed PAH concentrations between study sites reflected the variations in geographical location, proximity and influence of regional emissions, meteorology, and lake chemistry. Cummeenoughter is a high elevation mountain lake strongly influenced by incoming southwesterly air and high annual rainfall (>3000 mm). The soils at CUM are lithosols (shallow mineral soils) with sparse vegetation (e.g., bryophytes and graminoids) in areas. In contrast, the ADA and MUL study sites are both strongly influenced by their western exposure, as evidenced by elevated lake conductivity, Cl^−^ and Na^+^ owing to marine inputs. Further, soils at ADA and MUL are blanket peats overlain with *Calluna vulgaris* and graminoids, leading to high TOC and low pH in the study lakes. The eastern sites, SGI and CLE, are both more influenced by source emissions from Europe and Ireland based on back-trajectory analysis (see Figure 2).

 As such, SGI and CLE exhibited a stronger anthropogenic atmospheric pollution signal, as indicated by higher concentrations of nonmarine SO_4_^2−^ in lakes. Soils at both sites are also classified as blanket peat; however, vegetation in some areas of CLE is sparse owing to peat erosion.

### 3.2. PAHs in Lakes

Estimated PAH concentrations in lake water were three times higher at SGI (577.1 pg L^−1^; Figure 3), with the other study sites displaying a similar range of concentrations (125–153 pg L^−1^). A wide range in concentrations have been observed in other studies; for example, in the Neretva River in Bosnia, the concentration of 19 PAHs at the reference site was 160 pg L^−1^, while the river mouth exhibited levels up to 4000 pg L^−1^ [39]. Similarly, a total of 23 dissolved PAHs were measured in remote mountainous lakes [40, 41] in the Alps, Austria (350 ± 190 pg L^−1^, 2417 masl), in the Caledonian, Norway (560 ± 60 pg L^−1^, 723 masl), Pyrenees, Spain (270 ± 190 and 580 ± 200 pg L^−1^, 2240 masl), Tatra, Slovakia (3400 ± 400 pg L^−1^, 2057 masl). In the Himalayas, Nepal, ΣPAHs in alpine lakes (*n* = 7) was estimated to be 1900 ± 1860 pg L^−1^ (4890–5300 masl; [42]). In a small rural lake, Esthwaite Water, UK, 14 dissolved PAHs (141000 ± 84000 pg L^−1^; [43]). In Harbour estuary, NJ, USA, a total of 36 dissolved PAHs were measured (10713 ± 4674 pg L^−1^; [44]).

 Total PAHs in Irish lakes were comprised largely of Phenanthrene (~57%) and Fluoranthene (~22%; Table 2). Atmospheric concentrations of PAHs show higher proportions of Phenanthrene, compared with Fluorene and Fluoranthene, which is also consistent with dissolved lake samples in various mountainous lakes [40–42]. The HMW compounds are often bound to particulate (predominantly black carbon, or soot [1]) and, therefore, are more associated with soils or sediments rather than the water column and are less available for SPMD uptake; however, particle-bound PAHs will leach into surface waters via TOC transport from the catchment [1]. In addition, HMW compounds also have higher *K*_ow_ coefficients and experience longer sorption rates to reach equilibrium in SPMDs [45]. In contrast, LMW compounds are more susceptible to seasonality and temperature, possessing higher vapour pressures and lower *K*_ow_ coefficients, which increases solubility in water and bioavailability. This increased availability leads to higher exchange in media (i.e., water, air, sediments, etc.), which favours leaching and degradation via microbes or photooxidation compared with HMW compounds [40, 46].

 Estimated concentrations of HMW PAHs in the study lakes were negatively correlated with lake : catchment ratio (*R* = −0.87), highlighting increased inputs from relatively larger drainage areas, whereas LMW PAH estimates in lake water exhibit a positive correlation with 1-day overland ROI+NI air (*R* = 0.88), indicative of a potential source region originating from within Ireland, likely attributable to more local combustion sources. In addition, Naphthalene in lakes showed a negative correlation with TOC (*R* = −0.88) and SOM (*R* = −0.99), and a positive correlation with soil pH (*R* = 0.92) and annual rainfall (*R* = 0.98). The association with organic matter and relationship with precipitation underscores the elevated volatility and solubility coefficient (30.2 versus <3.93; [45]) of Naphthalene compared with “heavier” PAH compounds.

### 3.3. PAHs in Soils

Similar to lake water, the level of PAHs in soils was at least three-fold higher at SGI (1369 ng g^−1^ (109.5 *μ*g m^−2^)) compared with the other study sites, which ranged from 52.2–428.0 ng g^−1^ (9.20–25.7 *μ*g m^−2^; Figure 3). The concentrations were similar to grassland soils from a transect study between the UK and Norway (15 PAHs; 63 (Norway)–700 (UK) ng g^−1^; [47]), and on the lower end compared with concentrations observed in higher elevation European mountain ranges, which analyzed a greater number of PAHs in soils (Teide, Pyrenees, and Tatra; 23 PAHs between 750 and 2500 ng g^−1^: [48]). Soil organic carbon (OC) was high in the study sites (median: 47.3% OC) compared with other studies, as such, pool sizes were smaller. Alpine catchments observed mean PAH concentrations at Montseny 1000 ng g^−1^ (324 masl; 1.6% OC), Pyrenees 550 ng g^−1^ (1516 masl; 15.8% OC), Alps 1600 ng g^−1^ (1101 masl; 2.1% OC), Tatra 1400 ng g^−1^ (1413 masl; 12% OC [49]), and the Chilean Andes 600–4243 ng g^−1^ (1368 masl; [50]). Lower mean values for 16 PAHs measured in the Canadian Rocky Mountains, 68.3 ng g^−1^ [51] were more consistent with values representative of tropical regions (e.g., Costa Rica 1–36 ng g^−1^ [52]) rather than temperate background regions >200 ng g^−1^ [1]. 

 Total PAHs in the study soils were dominated by Fluoranthene (~31%) and Pyrene (~20%; Table 2). This proportional pattern was also observed in Montseny soils (394 masl; [49]) despite the substantial difference in SOM (2.8%, compared with average ~75% in this study). Soil properties can lead to major differences in PAH spatially and temporally; for instance, PAHs often undergo sorption to organic-rich substrate; however, particle size, polarity, soil pH, temperature, and moisture greatly affect their degradation and transport [1, 53]. In addition, total PAHs tend to be lower in mineral soils with a higher proportion consisting of HMW [54], which is consistent with observations in the mineral soils at CUM compared with the other organic-rich study catchments based on concentration per weight rather than pool size. Organic soils have a lower overall density but larger surface area, and the higher density of binding sites increases sorption capacity [46]. Further, PAHs with similar physiochemical properties are generally correlated [55], as observed in the study catchment (e.g., Fluorene and Anthracene (*R* = 0.90)). In contrast to water estimates of Naphthalene, concentrations in soils were positively correlated with TOC (*R* = 0.93) and negatively correlated with lake and soil pH (*R* = 0.88) and rainfall (*R* = −0.97), whereas Phenanthrene exhibited a negative correlation with soil pH (*R* = −0.88), related to organic acids, suggesting an association with SOM. Benz[a]anthracene+Chrysene was also correlated with lake : catchment ratio (*R* = −0.88), again demonstrating the influence of a relatively larger drainage basin.

### 3.4. Source Diagnosis

**Figure 4:**
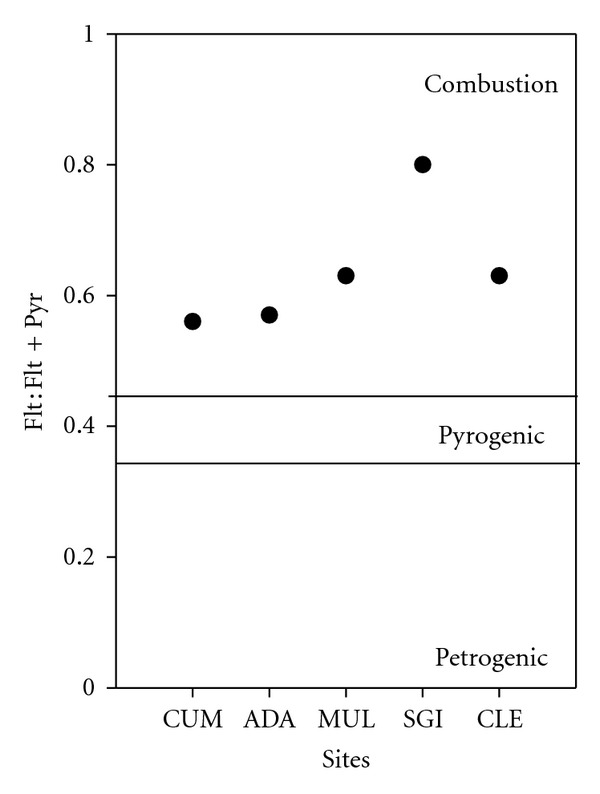
Ratios of Fluoranthene : Fluoranthene + Pyrene in soils and potential emission sources for the study catchments (Lough Cummeenoughter (CUM), Lough Adanacleveen (ADA), Mullincrick Lough (MUL), Sgilloge Lough (SGI), and Cleevaun Lough (CLE)).

Concentrations of PAHs in soils and estimated concentrations in water were highest at SGI, suggesting proximity to local emission sources in the east of the country as PAHs are produced primarily as a byproduct of various combustion processes. In contrast, the lowest soil concentrations were observed at CUM, suggesting the site predominantly received clean Atlantic air; further, the catchment is located in a mountainous region compared with SGI which is located in an agricultural region where vegetation burning is common. In general, PAHs typically have a lower volatility and therefore a shorter atmospheric residence time owing to their transport in association with particles [48]; as such, national emission sources potentially contribute the majority of these pollutants. Fluoranthene : Fluoranthene + Pyrene ratios (Figure 4) among sites were consistent with sources originating from combustion of grass, coal, or wood rather than fossil fuels (e.g., diesel or petroleum). Ratios <0.4 are considered petrogenic, while ratios between 0.4-0.5 are pyrogenic, and ratios >0.5 are attributed to combustion of organic matter [56]. Ratios provide an indication of source; however, they can vary greatly even from the same source owing to different atmospheric conditions [10]. Further, discrepancies in wind speeds, moisture, sunlight, and temperature among sites highly influence volatilization [57]. Correlations between LMW PAHs in lakes and soils and 1-day overland air originating from ROI and NI (*R* = 0.88 and 0.89, resp.) further suggest local combustion sources, supporting the higher concentrations measured in the eastern-southeastern sites. Back trajectory analysis showed higher proportions of 1-day overland air originating from ROI and NI at CLE (21.1%) and SGI (17.6%) compared with CUM (9.6%), ADA (9.2%), and MUL (10.9% (Figure 2 and Supporting Information Figure SI-1 available online at doi:10.1100/2012/828343)).

### 3.5. Principal Component Analysis

**Figure 5:**
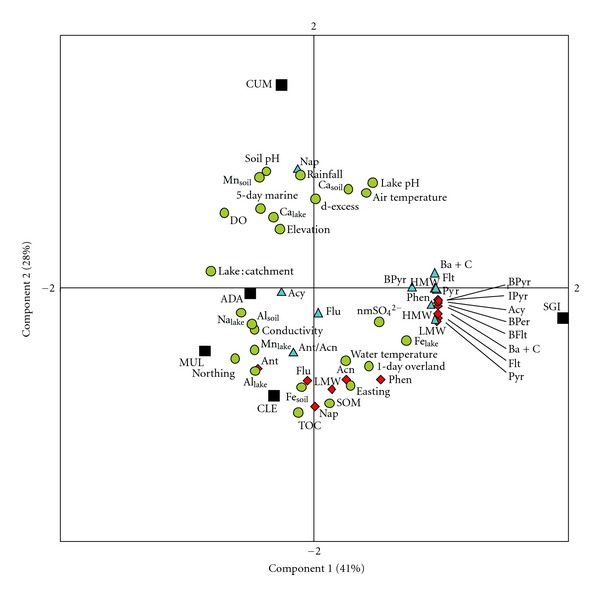
Principle component analysis (PCA) showing study catchments (black-filled square), lake characteristics (green-filled circles), lake PAH concentrations (blue-filled triangles), and soil PAH concentrations (red-filled diamonds). PC1 (41%), PC2 (28%), and PC3 ((not shown) 23%). Study sites: Lough Cummeenoughter (CUM), Lough Adanacleveen (ADA), Mullincrick Lough (MUL), Sgilloge Lough (SGI), and Cleevaun Lough (CLE).

The PCA (Figure 5) illustrated codistributed parameters (lake characteristics, and lake and soil PAH concentrations) and their relation to the five study sites. The ordination did not separate compounds with similar concentrations among sites; however, the ordination was strongly influenced by the high concentrations of some compounds at SGI. Component 1 (PC1), which described 41% of the variability in the parameters, was most highly influenced by Fe lake concentration (positively), and lake : catchment ratio and DO (negatively). Positive correlations with PC1 were observed for HMW compounds (soil and water) and LMW compounds (water). Component 2 (PC2), which described 28% of the variability in the parameters, was characterized by positive loadings for TOC, SOM, Al lake concentrations and Fe soil concentrations as well as negative loadings from pH (soil and water), and rainfall. Positive correlations with the PC2 axis were seen for LMW PAHs in soil (e.g., Naphthalene, Anthracene, and Fluorene), indicating their codistribution with organic matter and potentially reduced leaching rates with less precipitation. Component 3 (PC3, ~23% (not shown)), was most strongly influenced by the proportion of 1-day overland air, nonmarine SO_4_^2−^, and elevation (positively) and conductivity and water temperature (negatively). Acenaphthene, Fluorene, and Anthracene in lakes were positively correlated with this axis indicating their codistribution and association with the eastern-most sites (CLE and SGI) and therefore 1-day overland air, which was significantly correlated with anthropogenic emissions.

## 4. Conclusion

Although higher concentrations were observed at the southeastern-most site (SGI), the results suggest that upland seminatural headwater catchments in Ireland are representative of midlatitude background regions (>200 ng g^−1^; [1]), owing to the low levels of PAHs (solely from atmospheric deposition). Furthermore, PAH concentrations were well within, or below, the range of “background” values observed in continental Europe and internationally. Airshed movement and SOM strongly influenced the fate and transport of PAHs in headwater lakes in Ireland. Individual site physical, meteorological, and chemical parameters exhibited correlations with PAHs in SPMDs and soil samples, for example, lake : catchment ratio, rainfall, TOC, and source air, which highlighted the propensity of some compounds to washout, bind to organic matter, or undergo atmospheric transport. Further, Fluoranthene : Fluoranthene + Pyrene ratios suggested that wood, coal, and grass combustion were the primary source of PAH to the study sites. Media partitioning was evident in the study catchments; that is, even in highly organic soils, Naphthalene has a tendency to revolatilize or washout more readily and is more likely to be captured in SPMDs, whereas “heavier” HMW compounds bind to soil and sediments. 

 Although the responses of individual PAHs in water are well known [58], few studies have been conducted in Ireland. This study is the first catchment-based study to quantify PAHs in soils in combination with lake waters (using passive sampling techniques). It provides baseline data for future research and also provides a reference for other land-covers (e.g., urban, industrial, etc.). The vulnerability of PAHs to climate change should be investigated (i.e., [59, 60]). Increasing levels of DOC, altered hydrological processes, and land use changes may ultimately influence the release of long-term stores from organic-rich soils [61], which may have profound effects on PAH cycling in ecosystems. In particular, the breakdown of SOM leading to increased DOC in surface waters [62] will influence the remobilization of these compounds from their peaty reservoirs into the surrounding environment.

## Supplementary Material

Supplementary figure SI-1 illustrates an example of two-day back-trajectories (estimated every six hours) to Cleevaun Lough (CLE, see Figure 1 and Table 1) during the period December 2008 to November 2009; trajectories were allocated to four dominant clusters, closely representing cardinal directions (north [green], south [light blue], east [red] and west [blue]).

## Figures and Tables

**Table 1:** Chemical, physical, and meteorological characteristics of study catchments (*n* = 5): Lough Cummeenoughter (CUM), Lough Adanacleveen (ADA), Mullincrick Lough (MUL), Sgilloge Lough (SGI), and Cleevaun Lough (CLE). Lake chemical observations represent average concentrations during the period 2010-2011 (*n* = 6).

Site characteristics	CUM	ADA	MUL	SGI	CLE
Easting (m)	80300	91900	187600	229700	307100
Northing (m)	84800	314200	416700	111500	207300
Elevation (masl)	713.0	554.0	493.0	505.1	684.7
Lake size (ha)	0.35	1.52	0.85	2.36	1.78
Catchment size (ha)	1.90	15.5	4.50	63.4	10.1
% over land (1-day mean)	11.52	11.32	14.42	21.76	27.04
Water temperature (°C)	7.59	9.35	11.07	10.53	8.43
Rainfall (mm yr^−1^)	3060	1701	2003	1895	1847
Air temperature (°C)	10.28	9.30	9.02	9.80	9.59
Lake measurements					
pH	6.44	4.75	4.96	5.93	4.70
Conductivity (*μ*S cm^−1^)	24.95	56.46	57.89	29.79	26.00
Total organic carbon (mg L^−1^)	1.10	4.05	4.82	3.77	6.01
Gran alkalinity (mg L^−1^)	0.35	−0.75	−0.59	0.22	−0.66
Sulphate (mg L^−1^)	1.42	2.17	2.41	1.48	1.54
Chloride (mg L^−1^)	6.00	13.24	14.49	5.04	4.53
Dissolved oxygen (mg L^−1^)	11.88	11.84	11.53	11.19	11.49
Soil measurements					
Soil type	Lithosol	Peat	Peat	Peat	Peat
Bulk density (g cm^−3^)	0.54	0.13	0.14	0.16	0.12
pH (H_2_O)	4.35	4.01	4.00	3.83	3.85
Loss-on-ignition (%)	7.04	96.25	96.00	92.95	85.13
Nitrogen (%)	0.32	1.69	1.70	2.07	2.01
Carbon (%)	3.57	47.46	47.3	47.74	44.23
Sulphur (%)	0.05	0.34	0.30	0.36	0.37

**Table 2:** Estimated lake water and measured soil concentrations (0–5 cm) of 15 PAHs for upland study catchment (*n* = 5): Lough Cummeenoughter (CUM), Lough Adanacleveen (ADA), Mullincrick Lough (MUL), Sgilloge Lough (SGI), and Cleevaun Lough (CLE).

PAHs	Rings		Estimated lake concentrations (pg L^−1^)					Soil concentrations (ng g^−1^)				
	no.	Log *K*_ow_	Range					Range				
			Min	Max	Mean	SD	%ΣPAHs	Min	Max	Mean	SD	%ΣPAHs
Naphthalene	2	3.45	ND	4.55	0.91	2.03	0.40	6.57	15.9	12.8	3.66	2.92
Acenaphthylene	3	4.08	ND	11.0	2.21	4.94	0.97	0.00	3.36	0.67	1.5	0.15
Acenaphthene	3	4.22	ND	8.32	1.66	3.72	0.73	0.24	3.51	1.70	1.3	0.39
Fluorene	3	4.38	ND	12.8	6.79	4.84	2.98	0.45	12.4	5.20	4.5	1.19
Phenanthrene	3	4.46	65.3	302.0	129.0	98.7	56.7	2.91	91.6	47.4	37.7	10.8
Anthracene	3	4.54	ND	38.0	7.59	17.0	3.34	1.02	70.4	25.1	27.6	5.73
Fluoranthene	4	5.2	0.63	163.1	49.6	64.6	21.8	16.8	567.8	160.8	232.2	36.7
Pyrene	4	5.3	ND	92.6	25.3	38.0	11.1	13.5	272.3	84.8	108.1	19.4
Benz[a]anthracene+Chrysene*	4	5.61–5.91	ND	12.4	3.86	0.52	1.70	4.62	158.8	41.2	66.2	9.40
Benzo[b]fluoranthene*	5	5.78	ND	ND	ND	ND	0.00	1.92	169.1	38.3	73.2	8.76
Benzo[a]pyrene*	5	6.35	ND	1.21	0.47	0.52	0.21	ND	7.44	1.61	3.3	0.37
Benzo[ghi]perylene	6	6.75	ND	ND	ND	ND	0.00	ND	63.4	13.2	28.1	3.02
Dibenz[a,h]anthracene*	5	6.51	ND	ND	ND	ND	0.00	ND	ND	ND	ND	0.00
Indeno[1,2,3-cd]pyrene*	6	6.9	ND	ND	ND	ND	0.00	ND	24.5	4.89	10.9	1.12

*known carcinogen.
