# Supplementary material for: Fate and Transport of Polycyclic Aromatic Hydrocarbons in Upland Irish Headwater Lake Catchments
Source: ScientificWorldJournal. 2012 Dec 31;2012:828343. doi: 10.1100/2012/828343 (PMC3549342; doi:10.1100/2012/828343)
Supplement: Supplementary file 1 — Supplementary figure SI-1 illustrates an example of two-day back-trajectories (estimated every six hours) to Cleevaun Lough (CLE, see Figure 1 and Table 1) during the period December 2008 to November 2009; trajectories were allocated to four dominant clusters, closely representing cardinal directions (north [green], south [light blue], east [red] and west [blue]). [file 828343.f1.doc]

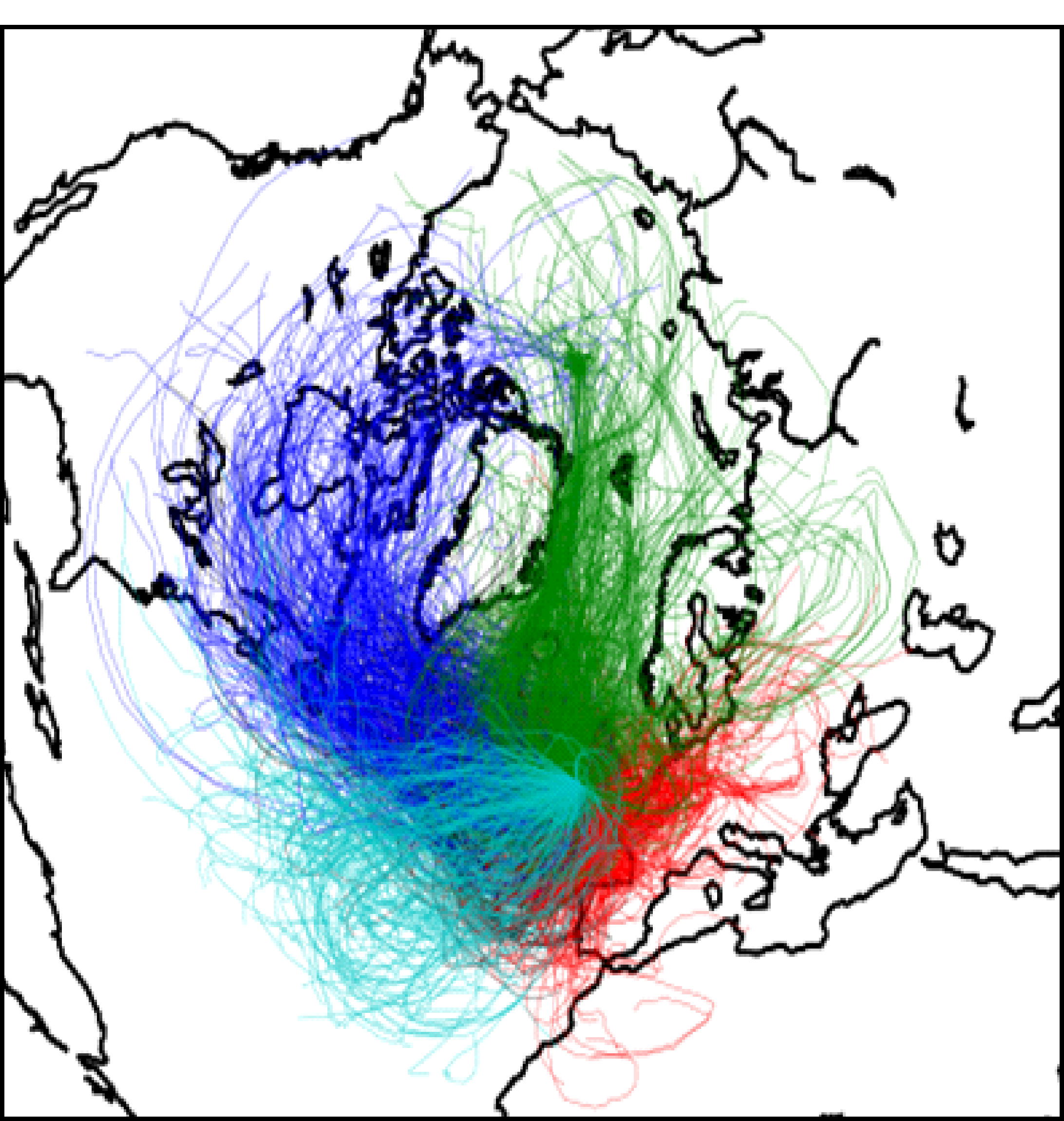


Figure SI-1. Example of two-day back-trajectories (estimated every six hours) to Cleevaun Lough (CLE, see Figure 1 and Table 1) during the period December 2008 to November 2009; trajectories were allocated to four dominant clusters, closely representing cardinal directions (north [green], south [light blue], east [red] and west [blue]).
